# Malnutrition impairs mitochondrial function and leukocyte activation

**DOI:** 10.1186/s12937-019-0514-7

**Published:** 2019-12-26

**Authors:** Celia Bañuls, Aranzazu M. de Marañon, Silvia Veses, Iciar Castro-Vega, Sandra López-Domènech, Christian Salom-Vendrell, Samuel Orden, Ángeles Álvarez, Milagros Rocha, Víctor M. Víctor, Antonio Hernández-Mijares

**Affiliations:** 1 Service of Endocrinology, Foundation for the Promotion of Health and Biomedical Research in the Valencian Region (FISABIO), University Hospital Doctor Peset, Avda Gaspar Aguilar 90, 46017 Valencia, Spain; 2 Institute of Health Research INCLIVA, University of Valencia, Valencia, Spain; 3 Department of Medicine, University of Valencia, Valencia, Spain; 4 CIBERehd - Department of Pharmacology and Physiology, University of Valencia, Valencia, Spain; 5 Department of Physiology, University of Valencia, Valencia, Spain

**Keywords:** Disease-related malnutrition, Oxidative stress, Outpatient population, Inflammation, Endothelial function, Cytokines

## Abstract

**Background:**

The aim of this study was to evaluate markers of inflammation, oxidative stress and endothelial function in a disease-related malnutrition (DRM) outpatient population.

**Methods:**

For this cross-sectional study, a total of 83 subjects were included and clustered in 3 groups: 34 with normonutrition (NN), 21 with DRM without inflammation (DRM-I) and 28 with DRM and inflammation (DRM + I). Nutritional diagnosis was conducted for all subjects according to ASPEN. Biochemical parameters, proinflammatory cytokines, reactive oxygen species production, glutathione, mitochondrial membrane potential, oxygen consumption, adhesion molecules and leukocyte-endothelium interactions were evaluated.

**Results:**

DRM + I patients showed lower albumin, prealbumin, transferrin, and retinol-binding protein levels with respect to the NN group (*p* < 0.05), differences that were less noticeable in the DRM-I group. DRM + I was associated with a significant increase in hsCRP and IL6 vs the NN and DRM-I groups, and TNFα was increased in both DRM vs NN. DRM was characterised by increased oxidative stress, which was marked by a significant increase in ROS levels and a decrease in mitochondrial membrane potential in the DRM + I group. An evident reduction in mitochondrial oxygen consumption and glutathione concentration was observed in both DRM groups, and was accompanied by increased leukocyte adhesion and adhesion molecules and decreased rolling velocity in the DRM + I group. Furthermore, percentage of weight loss was negatively correlated with albumin, prealbumin, transferrin, O_2_ consumption, glutathione and leukocyte rolling velocity, and positively correlated with hsCRP, IL6, TNFα, ROS, leukocyte adhesion, and VCAM-1.

**Conclusions:**

Our results show that DRM is associated with oxidative stress and an inflammatory state, with a deterioration of endothelial dysfunction in the DRM + I population.

## Background

Malnutrition refers to a state of nutrition in which there is an imbalance of energy, protein and other nutrients that causes adverse effects on the body and its functions [1]. The prevalence of malnutrition varies according to the population studied and the different methods used for screening, assessment and nutritional diagnosis. It is especially frequent in hospitalized populations and in the elderly due to associated health problems, while prevalence is reported to be lower (5–15%) among outpatients [2].

In the context of clinical nutrition practice, the term disease-related malnutrition (DRM) refers to a multifactorial condition due to a deficit of nutrients and triggered by increased nutrient loss, poor nutrient utilization and heightened nutritional requirements [3, 4]. DRM contributes to an inflammatory state due to the production of pro-inflammatory cytokines, which have an anorexiogenic effect on the patient and enhance protein catabolism [5, 6]. In this sense, the inflammatory response induced by the underlying disease represents a key factor for systemic low-grade inflammation and oxidative stress.

On the other hand, it has been demonstrated that malnutrition per se and DRM may involve insufficient intake of antioxidants and trace elements that are cofactors of antioxidant systems, as well as low levels of glutathione (GSH) [7, 8]. In this context, the malnutrition present during anorexia nervosa is characterised by mitochondrial dysfunction and oxidative stress in peripheral blood leukocytes at the level of the mitochondrial complex I [9]. During this process, immune cells, especially leukocytes, release proinflammatory cytokines and reactive oxygen species (ROS). Weight loss is mediated by some of these cytokines through various mechanisms, such as inefficient intake and a catabolic effect on energy reserves [6–8], and a positive association has been reported between these molecules and a low body mass index (BMI) [5, 6]. Moreover, research suggests that eating and swallowing difficulties, severe loss of appetite due to anorexiogenic effects of tumour necrosis factor-α (TNFα), high metabolic rate, loss of visceral proteins and reduction of fat-free mass together with oxidative stress can contribute to malnutrition and subsequent loss of weight [8, 10, 11]. Regarding the relationship between ROS and nutrition, it has been determined that there is a higher level of free radicals and lower antioxidant levels in protein-undernourished patients than in an adequately nourished population [7].

Proinflammatory cytokines, together with ROS, contribute to mitochondrial and endothelial dysfunction and oxidative stress [12]. In brief, malnutrition enhances ROS production, which activates inflammation pathways, and stimulates the production of adhesion molecules in endothelial cells. Hence, activated immune cells attach to the endothelium monolayer and infiltrate the inner organs. Several studies have confirmed this relationship. Our group have described an association between the reduced dietary intake in anorexia nervosa and an increase in leukocyte-endothelium interaction and, in turn, enhanced leukocyte migration [13]. This relationship between malnutrition and leukocytary activation has been also described by Stevinkel et al., who showed a strong relationship between undernourishment, inflammation and soluble adhesion molecule levels in pre-dialysis patients [14], a situation that could lead to increased leukocyte-endothelium interaction. A relationship between high levels of oxidative stress and leukocyte-endothelial adhesion has also been reported in animal models [15]. Based on this evidence, some studies have suggested that malnutrition is related to the development or aggravation of cardiovascular diseases (CVD) [16, 17].

Multiple definitions of malnutrition are used in the literature, which creates certain confusion. Recent evidence suggests that varying degrees of acute or chronic inflammation is a key contributing factor in the pathophysiology of DRM. An etiology-based approach that incorporates current understanding of the inflammatory response would seem to be appropriate. To enable this, the diagnosis of adult malnutrition in clinical settings according to the recent ASPEN and ESPEN consensus criteria [18] distinguishes between two types of DRM: DRM with inflammation (DRM + I), when malnutrition is associated with a chronic or acute inflammatory-based disease; and DRM without inflammation (DRM-I), found in starvation-related malnutrition. We hypothesized that DRM with or without inflammation produces an alteration of mitochondrial function that leads to oxidative stress, leading to an increase in leukocyte-endothelium interactions and endothelial dysfunction and favouring a chronic proinflammatory state, which is the basis of CVD. In addition, although few studies have assessed this topic in DRM, none of them have been performed in an outpatient population.

Therefore, the purpose of the present study was to identify possible differences between DRM-I and DRM + I with respect to proinflammatory state, oxidative stress and endothelial dysfunction markers in an outpatient population.

## Methods

### Subjects

The participants in this cross-sectional study consisted of outpatients attending the Endocrinology and Nutrition Service of the University Hospital Dr. Peset in Valencia (Spain) between January 2015 and December 2017.

Subjects aged 18 or older were eligible for inclusion in the study. Exclusion criteria were pregnancy or lactation, severe renal (chronic kidney disease in stage ≥4 and glomerular filtration rate < 30 mL/min) or severe hepatic disease (Child-pugh B or C), and previous monitoring by the Nutrition Unit. Patients were categorized based on the recent diagnostic consensus of malnutrition, employing the following etiology- based terminology: malnutrition without inflammation or DRM-I (starvation-related malnutrition); patients with anorexia; mechanic or neurologic dysphagia, in which inflammation was not the main cause of malnutrition; malnutrition with inflammation or DRM + I; and patients with chronic disease-related malnutrition and acute disease or injury-related malnutrition, specifically neoplasm, inflammatory bowel disease or chronic obstructive pulmonary disease (COPD), in which the disease and the chronic underlying inflammation played an active role in their nutritional status [18].

The study was conducted according to the ethical principles stated in the Declaration of Helsinki, and all procedures were approved by our hospital’s Ethics Committee. Written informed consent was obtained from all subjects.

### Assessment of nutritional status

A complete nutritional assessment was performed to evaluate the nutritional status of the subjects according to ASPEN criteria [4]. DRM was diagnosed when at least two of the following characteristics were confirmed: insufficient energy intake; weight loss; loss of muscle mass (wasting of the temples, clavicles, shoulders, interosseous muscles, scapula, thigh and calf); loss of subcutaneous fat (orbital, triceps, fat overlying the ribs); localized or generalized fluid retention; and decrease in functional status measured by gripping force (dynamometry).

For anthropometric determination, actual body weight and height without shoes were determined to the nearest 0.1 kg using an electronic scale and to the nearest 0.1 cm using a stadiometer, respectively. BMI was calculated from these results (BMI = weight in kg / (height in m) ^2^). To calculate the percentage of lost weight the following equation was applied: % PP = ((habitual weight - actual weight) / habitual weight) × 100.

Triceps skinfold thickness (TST) and mid-upper arm circumference (MUAC) were measured on the non-dominant arm and the mean of three measurements was calculated. TST was assessed using a skinfold calliper (Holtain LTD, Crymych, UK) and MUAC was determined at the middle point between the olecranon and acromion using a non-elastic tape measure. Arm muscle perimeter (AMP) was calculated using the formula AMP (Cm) = MUAC (cm) - (TST (mm) × 0.314). To prevent inter-observer and intra-observer variability, anthropometric parameters were collected by the same person.

### Blood sampling

Venous blood samples were obtained from the three groups of patients, after 12 h fasting. Centrifugation (1500 g, 10 min, 4 °C) of the samples was carried out for isolating serum and plasma in order to determine biochemical parameters. The leftover aliquots were stored at − 80 °C for other measurements.

### Biochemical determinations

All biochemical determinations were carried out in our hospital’s Clinical Analysis Service.

Albumin was determined by the Bromcresol green method (Abbott Laboratories, Abbott Park, IL 60064 USA) with a coefficient of variation (CV) ≤ 3.3% and a sensitivity of 0.3 g/dL. Prealbumin, transferrin, retinol-binding protein (RBP), and complement C3 fraction were determined by kinetic nephelometry with a Beckman LX-20 autoanalyzer (Beckman Coulter La Brea, CA, USA) with a CV of 4%. Triglycerides and total cholesterol were measured by enzymatic assays with a Beckman LX-20 autoanalyzer (Beckman Coulter, La Brea, CA, USA). The intraserial variation coefficient was < 3.5% for all determinations. Absolute lymphocytes were determined by flow cytometry (Coulter-Beckman) and high-sensitive C-reactive protein (hsCRP) by an immunonephelometric assay. The intraserial CV was < 3.5% for all determinations.

### Measurement of soluble proinflammatory cytokines and adhesion molecules

Serum levels of proinflammatory cytokines, interleukin-6 (IL6) and TNFα, and intercellular adhesion molecule 1 (ICAM-1) and vascular cell adhesion molecule 1 (VCAM-1) were analysed using a Luminex 200 flow analyser system (Austin, TX, USA). Milliplex® MAP human high sensitivity T Cell and Human Cardiovascular Disease Magnetic Bead Panel were purchased from Millipore Corporation (Billerica, MA, USA). The intraserial and interserial variation coefficients were < 5.0 and < 15.0%, respectively, for all determinations.

### Isolation of leukocytes

Citrated blood samples were mixed with dextran (3%) and left for 45 min in order to obtain human leukocytes. The supernatant was collected and poured over Ficoll-Hypaque (GE Healthcare, Uppsala, Sweden) and centrifuged at 1200 rpm for 25 min. The supernatant was discarded and the pellet was lysed for 5 min at room temperature using Lysis Buffer. The sample was then centrifuged (1200 rpm, 5 min) and the pellet washed in Hank’s Balance Salt Solution (HBSS) and resuspended in complete RPMI media (RPMI supplemented with 10% FBS).

### Measurement of oxidative stress parameters

Total ROS production, GSH content and membrane potential were determined using the fluorescent probes 2′, 7′-dichlorodihydrofluorescein diacetate (DCFH-DA, 5 μM) 5-chloromethylfluorescein diacetate (CMFDA, 2.5 μM) and tetramethylrhodamine methylester (TMRM, 5 μM), respectively. The leukocytes were seeded in 48-well plates and incubated with the probes for 30 min. Next, the wells were washed with HBSS and data was acquired with an IX81 Olympus fluorescence microscope (Olympus, Hamburg, Germany) and static cytometry software ‘ScanR’ version 2.03.2 (Olympus). Single cell fluorescence was measured and quantified. All probes were purchased from Invitrogen (Life Technologies, Barcelona, Spain). In order to assess Oxygen consumption, leukocytes were resuspended at a density of 5 × 10^6^ cells/mL in HBSS and placed in a gastight chamber coupled to a Clark-Type O_2_ electrode (Rank Brothers). In order to check whether O_2_ consumption was mainly mitochondrial, Sodium cyanide (10^− 3^ M) was employed.

### Adhesion assay

Umbilical cord samples obtained from normal deliveries were employed to harvest Human Umbilical Vein Endothelial Cells (HUVEC). In short, the procedure was as follows: the vein was washed with PBS and incubated in a solution of collagenase (1 mg/mL) for 17 min at 37 °C; after which the collagenase was collected in a 50 mL tube and neutralised with complete RPMI medium. The endothelial cells were then collected by centrifugation (1200 rpm, 10 min) and resuspended in Endothelial Growth Medium (EGM-2). The cells were seeded in T25 flasks and cultured until confluence. After confluence, cells were detached using trypsin and transferred to 6-well plates. The cells were cultured until confluence in 25 mm diameter plastic coverslips covered with fibronectin (5 mg/mL). In the flow chamber in vitro study, leukocytes (1·10^6^ cells/mL) were resuspended in Dulbecco’s PBS containing 20 × 10^− 3^ mol/L HEPES and 0.1% human serum albumin. The coverslip with confluent HUVEC monolayer was placed in the flow chamber and leukocytes were drawn across the monolayer at a flow rate of 0.36 mL/min (approximately shear stress of 0.7 dyne/cm^2^) under a microscope (Nikon Eclipse TE 2000-S; Amstleveen, The Netherlands) connected to a video camera (Sony Exware HAD; Koeln, Germany). Rolling and adhesion parameters in a single field were recorded for 5 min. Rolling velocity was evaluated by measuring the time in which a cell crossed a distance of 100 μm. Leukocyte rolling flux was determined by counting the number of leukocytes rolling over a surface of 100 μm^2^ of the endothelial monolayer during a 1-min period.

Adhesion was measured by counting the number of cells that maintained stable contact with the HUVEC monolayer for 30 s. The positive controls Platelet-activating factor (PAF, 1 μmol/L, 1 h) and TNFα, (10 ng/mL, 4 h) were used for leukocytes and HUVEC respectively.

### Statistical analysis

Based on our preliminary data [9, 12], the study was designed to detect 5 and 20% differences in variation of the serum cytokines (IL6 and TNFα) between and within groups, respectively, with a power of 80% and an α risk of 0.05. Under these premises, at least 20 subjects per group were considered.

The statistical program SPSS 17.0 was employed to perform the data analysis. The values in the tables are mean ± SD or median and 25th and 75th percentiles for parametric and non-parametric data, respectively. The bar graphs show mean ± SEM. The parametric data were compared with one-way analysis of variance (ANOVA), and the non-parametric data were compared with a Kruskal-Wallis test. Post-hoc tests were performed when needed (Student-Newman-Keuls or Dunn’s Multiple Comparison test for parametric and non-parametric data, respectively). In order to reduce the potential influence of age and gender analysis of covariance was employed. Correlations were calculated with Spearman’s correlation coefficient. Significant differences were considered when *p* < 0.05.

## Results

The present study analyzed a total of 83 outpatient subjects − 27 men and 56 women- with a mean age of 60.4 ± 17.5 years. Forty-nine of these patients were undernourished; 28 were diagnosed with DRM + I and 21 with DRM-I. The flow chart of the numbers of subjects throughout the study is shown in Additional file 1: Figure S1. Men and women were not represented similarly among properly nourished and undernourished subjects, and differences in age were detected between the study groups. The DRM group was characterized by the presence of neoplasm (38.8%), chronic renal disease (2.0%), COPD (6.1%), anorexia (22.4%), pneumonia (2.0%), digestive disease/Crohn’s disease (8.2%), ulcerative colitis (2.0%), pyloric stenosis (2.0%), choledocholithiasis (2.0%), rheumatic disesases (2.0%), circulatory diseases (4.0%) and neurological diseases (8.2%). The DRM + I group was comprised of patients with neoplasm, chronic obstructive pulmonary disease, ulcerative colitis, pneumonia, chronic renal disease and Crohn disease, while the DRM-I group included the rest of the patients.

As expected, baseline anthropometric characteristics differed between NN and DRM groups, with statistically significant differences observed regarding weight, BMI, percentage of weight loss, TST, MUAC and AMP, as shown in Table 1.

**Table 1:** Anthropometric, clinic and metabolic parameters in well nourished and disease-related malnutrition subjects according to the presence of inflammation

	NN (*n* = 34)	DRM-I (*n* = 21)	DRM + I (*n* = 28)	*p* value
Age (years)	60.0 ± 16.2	51.1 ± 23.5	65.8 ± 13.4^#^	0.030
Gender (% men)	23.5	14.3	57.1	0.002
Weight (Kg)	67.9 ± 15.6	45.8 ± 10.3***	58.8 ± 16.5*^##^	< 0.001
BMI (kg/m^2^)	25.8 ± 4.8	17.9 ± 4.2***	21.6 ± 4.8**^#^	< 0.001
Weight loss (%)	1.26 ± 5.10	13.8 ± 5.7***	17.1 ± 8.6***	< 0.001
Triceps Skinfold Thickness (mm)	19.3 ± 6.8	13.9 ± 6.2	14.3 ± 6.6*	0.009
Mid-upper arm circumference (cm)	28.7 ± 4.1	23.7 ± 3.5**	24.8 ± 4.3**	< 0.001
Arm Muscle Perimeter (cm)	22.6 ± 3.2	19.4 ± 2.9*	19.9 ± 3.3**	0.002
Albumin (g/dl)	4.22 ± 0.25	4.26 ± 0.43	3.62 ± 0.61***^###^	< 0.001
Prealbumin (mg/dl)	25.2 ± 4.6	24.9 ± 4.8	18.8 ± 6.8***^##^	< 0.001
Transferrin (mg/dl)	271.1 ± 54.5	231.8 ± 48.5	208.4 ± 66.8***	< 0.001
RBP (mg/dl)	3.65 ± 0.86	4.01 ± 0.82	2.95 ± 0.87*^#^	0.004
C3 (mg/dl)	104.5 ± 16.5	114.0 ± 23.4	113.8 ± 25.7	0.223
Total cholesterol (mg/dl)	193.2 ± 42.3	195.5 ± 43.8	172.3 ± 48.9	0.154
Triglycerides (mg/dl)	93.0 (67.8, 143.3)	92.0 (53.3, 115.5)	91.0 (79.0, 122.0)	0.308
Lymphocytes (× 109/l)	1.94 ± 0.61	1.92 ± 0.77	1.55 ± 0.80	0.152

Data are expressed as mean ± SD or median and 25th and 75th percentiles for parametric and non-parametric data, respectively. One-way analysis of variance of parametric data or Kruskal Wallis test for non-parametric data were employed to compare groups, followed by a post hoc test (Student-Newman-Keuls or Dunn’s Multiple Comparison test, respectively). * *p* < 0.05, **p < 0.01 and ***p < 0.001 vs NN; # *p* < 0.05 vs DRM-I; ##*p* < 0.01 vs DRM-I; ###*p* < 0.001 vs DRM-I

*DRM-I* Disease-related malnutrition without inflammation, *DRM + I* Disease-related malnutrition with inflammation, *NN* Normonutrition

In terms of metabolic parameters, DRM + I subjects displayed reduced albumin, prealbumin, and RBP with respect to the NN and DRM-I groups. In addition, DRM + I subjects showed decreased transferrin levels compared to the NN group. These statistically significant differences were not influenced by the age or gender of the subjects.

**Fig. 1:**
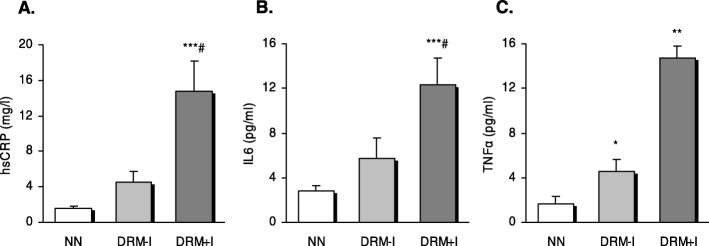
Proinflammatory cytokines in the serum of well nourished and disease-related malnutrition subjects according to the presence of inflammation. **a** Levels of hsCRP in serum (**b**) Levels of IL6 in serum (**c**) Levels of TNFα in serum. **p<0.01 and ***p<0.001 vs NN group, # *p*<0.05 vs DRM-I; using one-way ANOVA with Student–Newman–Keuls post-hoc test. DRM-I: disease-related malnutrition without inflammation; DRM+I: disease-related malnutrition with inflammation; NN: normonutrition.

Levels of inflammatory markers such as serum hsCRP, IL6 and TNFα were higher in the DRM + I group than in the NN (Fig. 1) and DRM-I (only for hsRP and IL6) groups. Surprisingly, DRM-I and DRM + I groups showed increased levels of TNFα with respect to NN subjects, with the difference with DRM + I subjects being more marked. Despite an increase of TNFα in the DRM + I vs. DRM-I group (evident in the graph), the difference was not statistically significant. These differences remained after adjustment for age and gender.

**Fig. 2:**
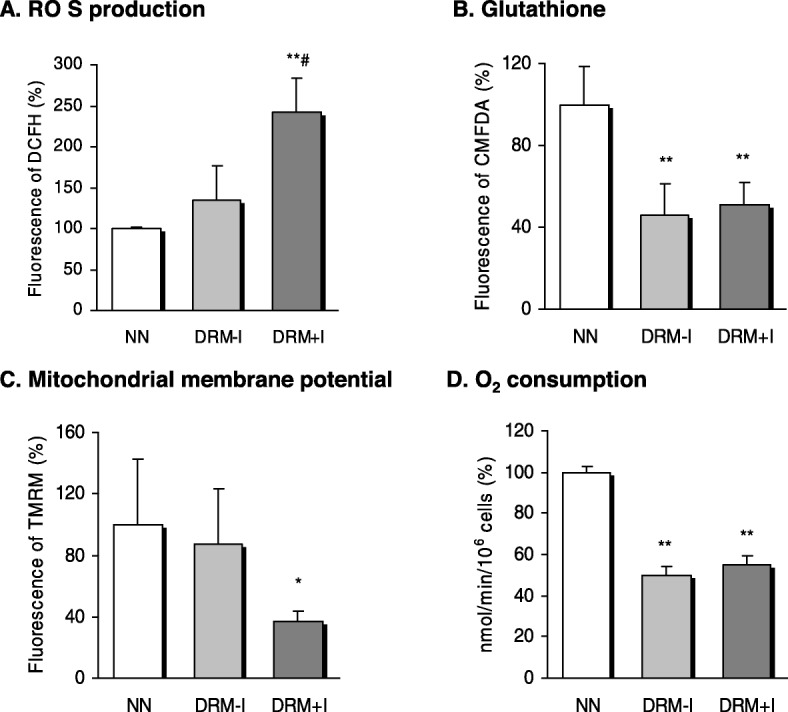
Oxidative stress parameters in the leukocytes of normonutrition and disease-related malnutrition subjects according to the presence of inflammation. **a** Levels of total ROS measured by DCFH-DA fluorescence and expressed as percentage of control (**b**) Levels of glutathione measured by CMFDA fluorescence and expressed as percentage of control (**c**) Levels of mitochondrial membrane potential measured by TMRM and expressed as percentage of control (**d**) Oxygen consumption. DRM-I: disease-related malnutrition without inflammation; DRM + I: disease-related malnutrition with inflammation, DCFH-DA: 2′,7′-dichlorodihydrofluorescein diacetate; CMFDA: 5-chloromethylfluorescein diacetate; NN: normonutrition; TMRM: tetramethylrhodamine methylester. *p < 0.05 and **p < 0.01 vs NN group, # *p* < 0.05 vs DRM-I using one-way ANOVA with Student–Newman–Keuls post-hoc test

Total ROS production was significantly higher in the DRM + I group than in the NN and DRM-I groups. (Fig. 2a, *p* < 0.01). Furthermore, GSH levels (CMFDA) were significantly reduced in leukocytes of patients in both DRM groups (Fig. 2b, p < 0.01), and mitochondrial membrane potential (TMRM) fluorescence revealed lower levels in DRM + I patients with respect to NN subjects (Fig. 2c, *p* < 0.05). In addition, mitochondrial oxygen consumption was lower in both DRM groups than in the NN group (Fig. 2d, *p* < 0.01).

**Fig. 3:**
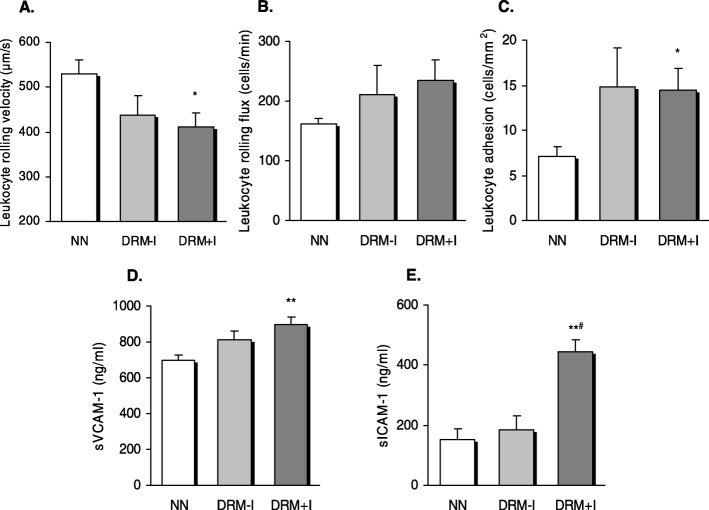
Leukocyte/endothelium interactions and serum soluble cell adhesion molecules levels in normonutrition and disease-related malnutrition subjects according to the presence of inflammation. **a** Leukocyte rolling velocity (μm/sec), **b** rolling flux (Cells/ min) and **c** Leukcoyte adhesion (Cells/mm^2^), **d** VCAM-1 levels, **e** ICAM-1 levels. *p < 0.05 and **p < 0.001 vs NN group, #*p* < 0.05 vs DRM-I using one-way ANOVA with Student–Newman–Keuls post-hoc test. DRM-I: disease-related malnutrition without inflammation; DRM + I: disease-related malnutrition with inflammation; NN: normonutrition.

Leukocyte rolling velocity (Fig**.** 3a) and leukocyte adhesion (Fig. 3c) were enhanced in DRM + I subjects with respect to their controls (*p* < 0.05). No significant differences were obtained between the effects of NN or DRM on leukocyte rolling flux (Fig. 3b). In addition, an increase in ICAM-1 and VCAM-1 were detected in DRM + I subjects with respect to the NN group (*p* < 0.05) (Fig. 3d and e).

We explored potential correlations among biochemical, inflammatory and oxidative stress markers and found that percentage of weight loss was negatively correlated with albumin (*r* = − 0.335, *p* = 0.005), prealbumin (*r* = − 0.417, *p* = 0.001), transferrin (*r* = − 0.387, *p* = 0.002) leukocyte rolling velocity (*r* = − 0.440. p = 0.001), glutathione (*r* = − 0.500, *p* = 0.015) and O2 consumption (*r* = − 0.403, *p* = 0.010), and positively correlated with hsCRP (*r* = 0.451, *p* < 0.001), IL6 (*r* = 0.575, *p* < 0.001), TNFα (*r* = 0.517, *p* = 0.001), DCFH (*r* = 0.490, *p* = 0.001), leukocyte adhesion (*r* = 0.296, *p* = 0.033) and VCAM1 (*r* = 0.543, *p* < 0.001). In addition, albumin was negatively correlated with hsCRP (*r* = − 0.449, *p* < 0.001), IL6 (*r* = − 0.296, *p* = 0.033), DCFH (*r* = − 0.338, *p* = 0.015) and positively correlated with glutathione (*r* = 0.402, *p* = 0.030) and ICAM1 (*r* = − 0.513, *p* < 0.001), while prealbumin was negatively correlated with hsCRP (*r* = − 0.308, *p* = 0.015), TNFα (*r* = − 0.359, *p* = 0.012), VCAM-1 (*r* = − 0.295, *p* = 0.044) and DCFH (*r* = − 0.317, *p* = 0.025). Finally, transferrin was negatively correlated with hsCRP (*r* = − 0.379, *p* = 0.002) and DCFH (*r* = − 0.310, *p* = 0.027).

## Discussion

The present study provides evidence that DRM, associated or not with inflammation, can result in a reduction of antioxidant molecules and mitochondrial oxygen consumption. Specifically, DRM + I subjects display enhanced inflammation parameters and production of pro-oxidant molecules, which undermine leukocyte-endothelium interaction and mitochondrial function.

Regarding anthropometric measurements, the present data demonstrate that BMI, TST, MUAC, and AMP have a significant impact on DRM patients. These findings are in line with those of previous studies [19, 20] which suggested that DRM is associated with a reduction in anthropometric measurements. However, these previous studies proposed a relationship with a reduction in anthropometrical parameters that depended on the inflammatory status. The present results demonstrate that the relationship between the reduction in anthropometric parameters and the presence of DRM does not depend on inflammatory status, as only biochemical parameters are significantly altered when inflammation is present. Specifically, DRM + I subjects show distinctive alterations of biochemical malnutrition markers (albumin, prealbumin, transferrin and RBP).

It is well known that non-nutritional factors affect biochemical parameters, but variations of the nutritional state also induce these changes [21, 22]. Therefore, in order to assess nutritional status, the parameters associated with the visceral protein compartment were evaluated, taking into account that an increase of acute phase reactants correlates negatively with these parameters. Our results reveal significant alterations in acute phase reactants only in the DRM + I group. Specifically, albumin has traditionally been known as a nutritional marker. In the present study, we found that DRM + I patients had significantly lower albumin levels than NN and DRM-I subjects, which confirms that this molecule acts as a marker only when inflammation is present. In addition, a drop in prealbumin and RBP was observed in the DRM + I group, thus confirming that these molecules are important markers of DRM + I, but not of DRM-I. Moreover, the observed increase in the level of proinflammatory cytokines could have been related to the increase in acute reactant proteins in our DRM + I subjects. This relationship has previously been assessed in chronic kidney disease and in haemodialysis patients [23, 24]. In short, albumin and RBP are markers only when inflammation is present, and so are not reliable markers in DRM-I patients, which reflects the importance of inflammatory status in DRM patients.

It is widely known that inflammation is an important factor affecting DRM patients, as this study confirms. In this sense, Meuwese et al. reported that inflammation induced an increase of lipolysis and muscle protein impairment - leading to sarcopenia, anorexia and increased mortality - in patients with chronic kidney disease [23]. In addition, some studies have shown higher CRP levels in haemodialyzed malnourished patients vs. NN subjects, which is a risk factor for cardiovascular diseases [16, 17, 19]. Our findings confirm that hsCRP correlates positively with weight loss, which implies a worsening of the inflammatory state in both types of DRM patients. Moreover, inflammatory markers such as IL6 or TNFα are enhanced in haemodialysis patients with or without appetite loss [24, 25], and in anorexia nervosa patients [6, 13]. The present data demonstrate an increase in levels of IL6, hsCRP and TNFα in DRM + I patients, and an increase in levels of TNFα in DRM-I patients. The increase in proinflammatory cytokines correlated positively with both the percentage of weight loss and VCAM-1 levels. This data, together with that of the biochemical parameters assessed, confirm a distinctive pattern of markers in DRM + I patients that allow them to be differentiated from DRM-I subjects. Moreover, this highlights the molecular differences between DRM-I and DRM + I patients.

DRM samples exhibited an increased burden of oxidative stress with respect to NN subjects. Specifically, the DRM + I group displayed a rise in ROS and a reduction in mitochondrial membrane potential, whilst both DRM groups showed lower GSH levels and oxygen consumption. The underlying inflammation in the DRM + I group may have caused mitochondrial dysfunction, which would have been less pronounced in the DRM-I group. Several studies have related malnutrition with a rise of ROS in different types of diseases [11, 26–28]. In this sense, Hsu et al. reported high levels of ROS and altered mitochondrial function in subjects with folate deficiency, which suggests that mitochondrial dysfunction is a result of malnutrition [29]. Some research suggests that resolving malnutrition or supplementing patients with nutrients can resolve or reduce the associated rise in ROS [15, 29]. Moreover, our study is in accordance with others showing elevated levels of lipid peroxidation products, such as MDA, and decreased levels of antioxidants, such as glutathione peroxidase and catalase, in patients with chronic obstructive pulmonary disease versus controls [30, 31]. In this way, our results confirm that DRM patients have higher levels of ROS and that this leads to a proinflammatory status, a trend that is strongly marked in the DRM + I population. Mitochondrial function in the population studied was more negatively affected than in those without chronic inflammation.

Inflammation is an active agent in the progression of atherosclerosis [32, 33], and chronic inflammation can contribute to malnutrition. Stenvinkel et al. reported that inflammation and malnutrition are closely associated with arteriosclerosis/CVD, leading them to coin the term “malnutrition- inflammation-atherosclerosis (MIA) syndrome” [34]. According to their study, inflammatory cytokines play important roles in MIA syndrome, which may be an important cause of atherosclerosis and malnutrition. An enhancement of inflammatory markers and ROS could have caused the rise in leukocyte-endothelial interaction parameters detected in our study, such as leukocyte adhesion. We have also observed that leukocyte rolling velocity was negatively correlated with ROS production. Soluble cytokines produced by mononuclear leukocytes affect the surrounding endothelium, which expresses adhesion molecules, enabling the leukocyte to roll and migrate to the site of inflammation. In a state of chronic inflammation, this situation is enhanced. In malnourished dialysis patients a rise in adhesion and inflammatory molecules has been reported [14], and the present data confirm a rise in VCAM-1 and ICAM-1 levels in these conditions. The aforementioned group also correlated hsCRP levels with soluble ICAM-1, a result that the present study also corroborates.

This study has several limitations. The sample size is small, basically because the prevalence of malnutrition in our outpatient population is only around 5% [35]. It is difficult to compare our results with those of other studies, as little research has been carried out in DRM patients, and the studies in question have not assessed inflammation parameters. Thus, our results are of interest because of the novelty of the type of sample and our etiology-based approach, which has been devised to incorporate current understanding of the inflammatory response. Nevertheless, DRM include a diverse array of chronic diseases with that vary extensively in etiology and pathology. It would be relevant to clarify whether malnutrition is the cause of the changes we have observed rather than the disease itself. Future investigations should address these different populations in order to determine the pathophysiological mechanisms underlying malnutrition, to assess nutritional status and propose recommendations for the prevention and treatment of these patients.

## Conclusions

The present study provides a better understanding of the ongoing pathophysiological mechanisms at play in the leukocytes of DRM patients. They suggest that adverse mechanisms, by which DRM can modify oxidative status and mitochondrial function, induce leukocyte-endothelium interactions and moderate the inflammatory response in the systemic circulation, with DRM + I populations being affected more severely.

## Supplementary information

**Additional file 1: Figure S1.** Flow Chart of the number of subjects throughout the study.

## Data Availability

All data generated or analysed during this study are included in this published article.

## Abbreviations

AMP: Arm Muscle Perimeter
BMI: Body Mass Index
COPD: Chronic Obstructive Pulmonar Disease
CV: Coefficient of variation
CVD: Cardiovascular Diseases
DCFDA: 2′, 7′-dichlorodihydrofluorescein diacetate
DRM + I: DRM with inflammation
DRM: Disease-Related Malnutrition
DRM-I: DRM without inflammation
EGM-2: Endothelial Growth Medium 2
GSH: Glutathione
HBSS: Hank’s Balanced Salt Solution
HEPES: 4-(2-hydroxyethil)-1-piperazineethanesulfonic acid
hsCRP: High sensitivity C-reactive protein
HUVEC: Human Umbilical Vein Endothelial Cells
ICAM-1: Intercelular Adhesion Molecule 1
IL6: Interleukin 6
MUAC: Mid-Upper Arm Circumference
NN: Normonutrition
PBS: Phospate Buffered Saline
RBP: Retinol-binding protein
ROS: Radical Oxigen Species.
RPMI: Roswell Park Memorial Institute medium.
TMRM: Tetramethylrhodamine methylester
TNFα: Tumor Necrosis Factor alpha
TST: Triceps Skinfold Thickness
VCAM-1: Vascular Cell Adhesion Molecule 1
