# Supplementary figures and images for: Malnutrition impairs mitochondrial function and leukocyte activation
Source: Nutr J. 2019 Dec 26;18:89. doi: 10.1186/s12937-019-0514-7 (PMC6933906; doi:10.1186/s12937-019-0514-7)

## Participant FlowChart

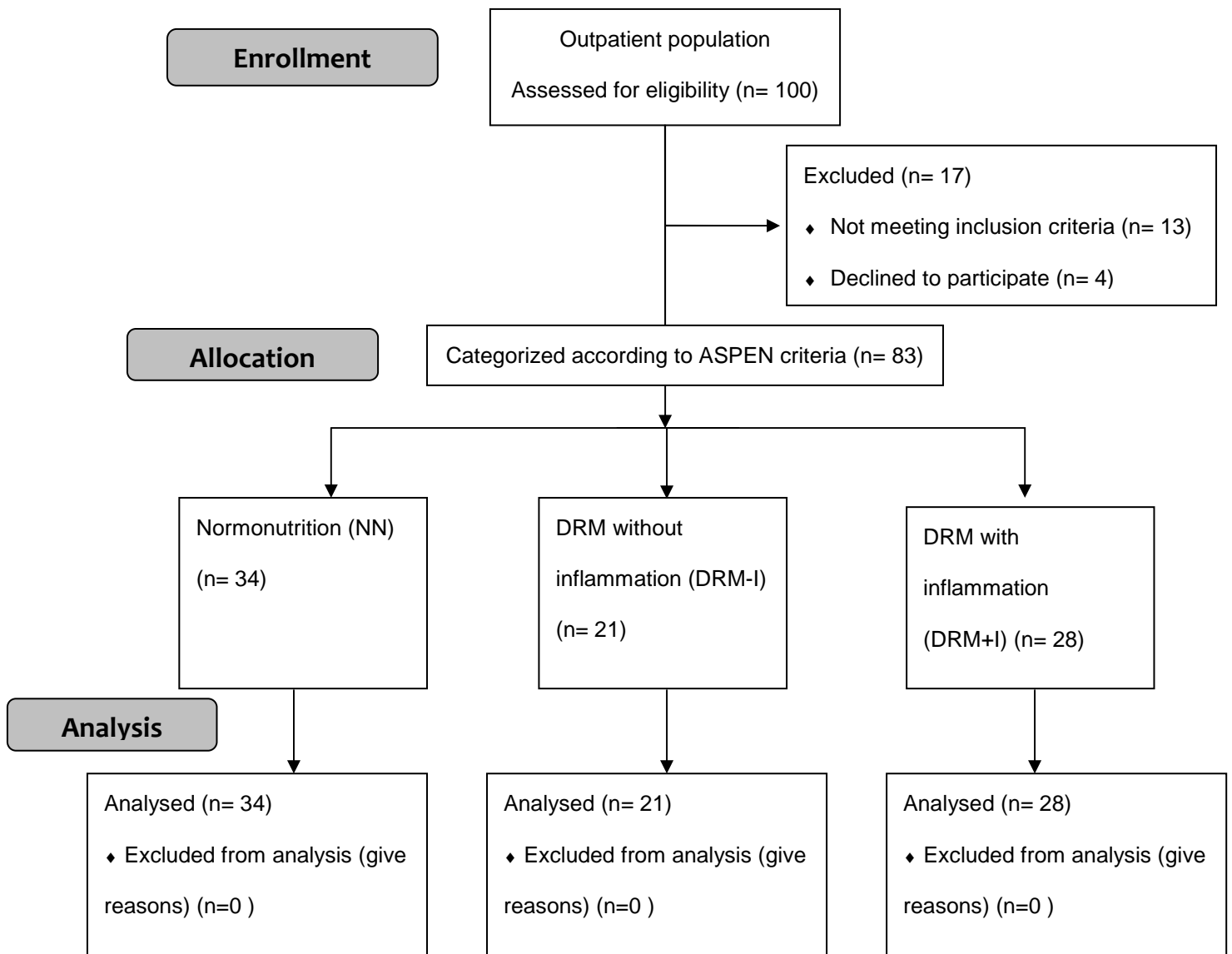

Supplement: Supplementary file 1 — Additional file 1: Figure S1. Flow Chart of the number of subjects throughout the study. [file 12937_2019_514_MOESM1_ESM.pdf]
